# A Stable Fe-Zn Modified Sludge-Derived Biochar for Diuron Removal: Kinetics, Isotherms, Mechanism, and Practical Research

**DOI:** 10.3390/molecules28062868

**Published:** 2023-03-22

**Authors:** Yucan Liu, Xianguo Ji, Ying Wang, Yan Zhang, Yanxiang Zhang, Wei Li, Jiang Yuan, Dong Ma, Hongwei Sun, Jinming Duan

**Affiliations:** 1 School of Civil Engineering, Yantai University, Yantai 264005, China; 2 School of Environmental and Materials Engineering, Yantai University, Yantai 264005, China; 3 Key Laboratory of Northwest Water Resources, Environment and Ecology, Ministry of Education, Xi’an University of Architecture and Technology, Xi’an 710055, China; 4 College of Environmental Science and Engineering, Tongji University, Shanghai 200092, China; 5 Rural Environmental Engineering Center of Qingdao, College of Resource and Environment, Qingdao Agricultural University, Qingdao 266109, China; 6 Centre for Water Management and Reuse, University of South Australia, Mawson Lakes Campus, Adelaide, SA 5095, Australia

**Keywords:** sludge-derived biochar, Fe-Zn modification, diuron, adsorption kinetics, adsorption isotherms, adsorption mechanism, practical research

## Abstract

To remove typical herbicide diuron effectively, a novel sludge-derived modified biochar (SDMBC600) was prepared using sludge-derived biochar (SDBC600) as raw material and Fe-Zn as an activator and modifier in this study. The physico-chemical properties of SDMBC600 and the adsorption behavior of diuron on the SDMBC600 were studied systematically. The adsorption mechanisms as well as practical applications of SDMBC600 were also investigated and examined. The results showed that the SDMBC600 was chemically loaded with Fe-Zn and SDMBC600 had a larger specific surface area (204 m^2^/g) and pore volume (0.0985 cm^3^/g). The adsorption of diuron on SDMBC600 followed pseudo-second-order kinetics and the Langmuir isotherm model, with a maximum diuron adsorption capacity of 17.7 mg/g. The biochar could maintain a good adsorption performance (8.88–12.9 mg/g) under wide water quality conditions, in the pH of 2–10 and with the presence of humic acid and six typical metallic ions of 0–20 mg/L. The adsorption mechanisms of SDMBC600 for diuron were found to include surface complexation, π–π binding, hydrogen bonding, as well as pore filling. Additionally, the SDMBC600 was tested to be very stable with very low Fe and Zn leaching concentration ≤0.203 mg/L in the wide pH range. In addition, the SDMBC600 could maintain a high adsorption capacity (99.6%) after four times of regeneration and therefore, SDMBC600 could have a promising application for diuron removal in water treatment.

## 1. Introduction

Diuron, a typically substituted urea herbicide, causes plants to wither by inhibiting photosynthesis. It is widely employed for the treatment of gramineous and some broad-leaved grasses [1]. Due to its complex and stable structure and long half-life, diuron is often detected in the effluent from municipal sewage treatment plants, surface water, and groundwater at different concentrations [2]. For example, diuron has been detected in UK (6742 ng/L) [3], Brazil (50–7800 ng/L) [4], and Japan (2.18 μg/L) [5]. Diuron has been characterized as a potential mutagenic/carcinogenic chemical by the United States Environmental Protection Agency (US EPA) [6]. Previous research has revealed the carcinogenic effect of diuron on rats as well as the cytotoxicity and potential genotoxicity on humans [7]. In addition, due to its increasing threat to human health, diuron was added to the priority list of pollutants for the water policy area in the European Commission, and the European Union has set the maximum allowable concentration of diuron in drinking water at 0.1 μg/L [8]. In this context, it is essential to adequately treat diuron-contaminated water.

Currently, the methods employed to treat diuron in water comprise the advanced oxidation process (AOPs), biological methods, membrane filter methods, and adsorption [9,10,11,12]. Although AOPs have shown good removal efficiency, the high energy and catalyst consumptions make this treatment method difficult to apply in practical water treatment engineering projects [13]. Although the biological methods can effectively remove diuron in water, water solution conditions (such as temperature and pH) affect adversely the effectiveness [9]. The membrane filter method has a high removal rate for diuron, but the high costs and issues with concentrated water of membrane filtration make it largely impractical [14]. As an efficient and environmentally friendly method [15], the adsorption method requires an expensive adsorbent, which limits its use [16]. Research for environmentally friendly, economic, sustainable, and highly efficient adsorbent is of importance [17].

Municipal excess activated sludge is a major solid waste of municipal sewage treatment. With the advancement of the world economy and urbanization, the excess activated sludge of urban sewage treatment plants increases year by year. At present, the excess activated sludge is generally treated by landfill or incineration. Such a treatment is prone to cause resource waste and secondary pollution [18], and is not in line with the global conception of carbon reduction and resource utilization of waste. Instead, the excess activated sludge is a kind of waste biomass material containing organic matter and nutrients as well as certain metal salts. Thus, the excess activated sludge can be potentially used for producing biochar. Using the residual activated sludge to prepare sludge biochar (SDBC) as an adsorbent has the advantages of waste reuse and high cost-efficiency. This can resultantly reduce the secondary pollution of the excess activated sludge and improve environmental safety. Studies of SDBC for removing organic pollutants from water have been reported recently. For example, the adsorption of atrazine by SDBC was studied [19]. However, the original SDBC had limited adsorption performance for atrazine because of its low number of functional groups, lack of pore capacity, and small specific surface area.

Studies on sludge-modified biochar have also been reported but with only limited success. For example, Liu et al. [20] used magnetic biochar prepared by co-pyrolysis of zero-valent iron nanoparticles and sewage sludge to remove Cr(VI) from water, with an adsorption capacity of 11.56 mg/g. Therefore, it would be useful if a better modification method could be established. Modification by metal ions is a common method of biochar modification. For example, Iron(III) is one of the most widely used media for modifying biochar because it increases the specific surface area and facilitates magnetic separation [21,22]. A study showed that the adsorption capacity of Fe-modified lignin biochar for removing methylene blue in wastewater could be 2.7 times greater than that of the unmodified original lignin biochar [23]. In addition, zinc salts were also found to be effective and low-cost modifiers for improving the pore structure and increasing the specific surface area of biochar for enhanced micropollutants adsorption [24]. In a previous study, the adsorption property of the bamboo biochar modified with ZnCl_2_ was tested to be 4.4 times greater than that of the original bamboo biochar [25]. The metal modification enables biochar to obtain more adsorption sites and a larger surface area, which solves the effective recovery of biochar after adsorption. However, the metal modification will cause the exudation of metal ions of biochar and pollute water. Moreover, Fe-Zn modified biochar could combine the advantages of two kinds of modified material, which is an effective adsorbent to remove organic synthetic raw material (P-nitrophenol [26]), heavy metals (Pb(II) [27], Cd(II) [28]), and antibiotics (tetracycline [29]) from wastewater. Additionally, bimetallic modification can improve the safety of biochar and reduce the leakage of metal ions. However, the preparation of sludge-derived modified biochar (SDMBC) using Fe-Zn has not been reported, and the adsorption efficiency and mechanism of diuron from water by SDMBC are unclear.

Therefore, the present study aimed at preparing a novel and efficient Fe-Zn modified sludge biochar for the adsorption of diuron in an aqueous solution. The main objectives of the study were to (1) prepare SDMBC by using SDBC as raw material and Fe-Zn as an activator and modifier; (2) analyze the adsorption kinetics and isotherms of diuron by SDMBC600 (prepared at 600 °C); (3) investigate the influence of solution conditions on the adsorption capacity of diuron by SDMBC600 and the underlying adsorption mechanisms; and (4) conduct practical research on SDMBC600 (pesticide mixture, real water, stability, and regeneration).

## 2. Results and Discussion

### 2.1. Biochar Characterization

**Figure 1:**
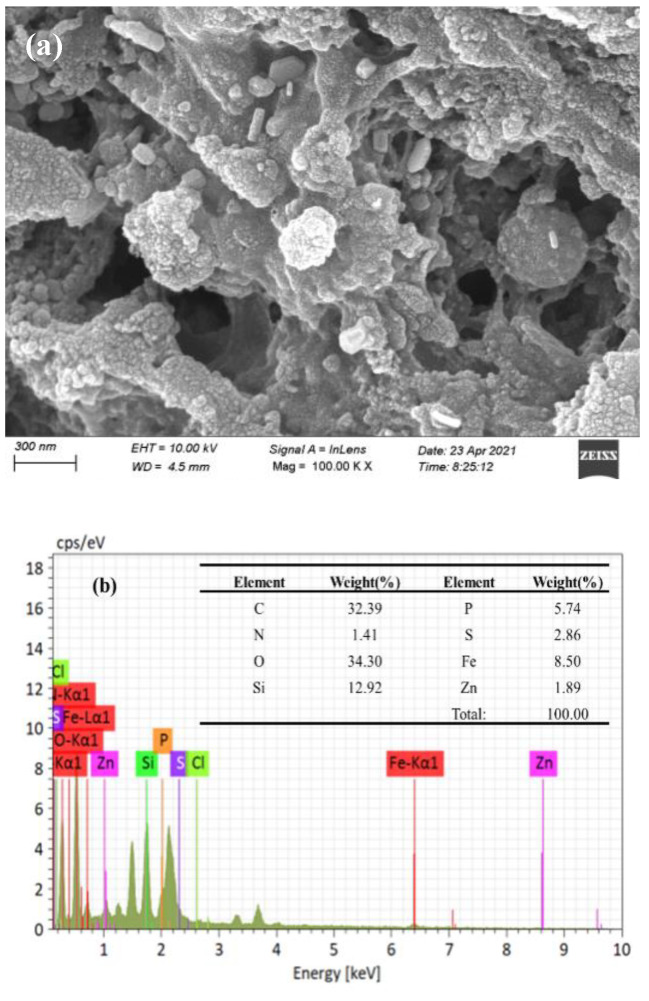

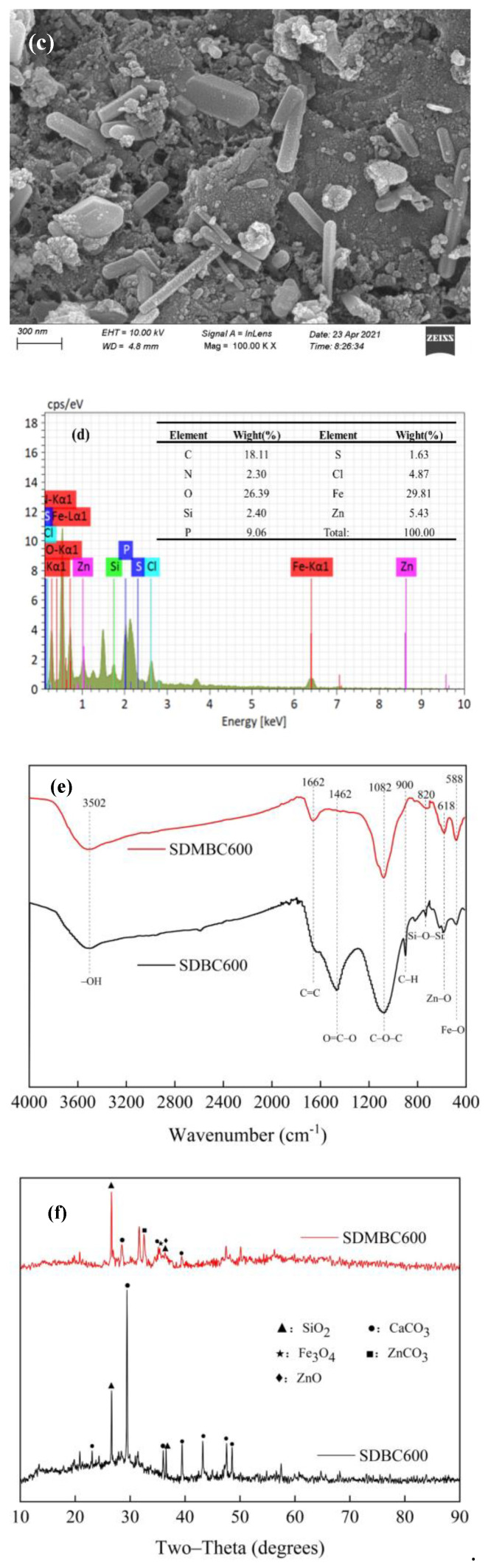
Biochar characterization: (**a**,**c**) the SEM images of SDBC600 and SDMBC600; (**b**,**d**) the EDS spectra of SDBC600 and SDMBC600; (**e**) the FTIR spectra of SDBC600 and SDMBC600; (**f**) the XRD diffraction pattern of SDBC600 and SDMBC600.

SEM was used for analyzing the surface morphology of SDBC600 and SDMBC600 (see Figure 1a,c). The SDBC600 had an irregular surface and a highly porous structure, providing loading space for Fe-Zn oxides (Fe_3_O_4_, ZnCO_3_, and ZnO). Compared with SDBC600, there were some short rods and polygonal columnar particles on the surface of SDMBC600, likely resulting from the loading of Fe-Zn oxide on the surface of the biochar. The EDS analysis proved that SDMBC600 was successfully loaded with Fe-Zn (see Figure 1b,d).

BET was used to determine the N_2_ adsorption-desorption curves of SDMBC600 (see Table S1 and Figure S1). The N_2_ adsorptions-desorption isotherm of SDMBC600 conformed to type IV adsorption isotherm following the classification of the International Union of Pure and Applied Chemistry (IUPAC) [30]. This indicates that SDMBC600 was mesoporous. Based on pore size distribution, the SDMBC600 had an average pore size in the range of 2−50 nm, further confirming its mesoporous characteristics [24]. The total pore volume of SDMBC600 (0.0985 cm^3^/g) was 2.58 times that of SDBC600 (0.0382 cm^3^/g). This may be due to the effect of ZnCl_2_ on the pore formation during carbonization, which increased the pore volume of the modified biochar [24]. SDMBC600 (204 m^2^/g) had a higher Langmuir surface area than SDBC600 (59.8 m^2^/g), this might be due to the loading of Fe-Zn oxides [28].

The polarity and carbonization degree of biochar could be reflected by its elemental composition [31]. The H/C ratio raised from 0.116 for SDBC600 to 0.166 for SDMBC600, while the content of C decreased from 22.98% for SDBC600 to 20.62% for SDMBC600 (Table S2). The carbonization degree of biochar could be evaluated by its H/C [32]. The H/C data indicated that SDMBC600 was less carbonized than SDBC600 in the study. Generally, (N+O)/C could be applied to assess the numbers of polarity groups in biochar. If the (N+O)/C ratio of biochar was low, this indicates that its polarity was low and aromatic hydrocarbons were strong [31]. In addition, O/C could be employed to estimate the hydrophilicity of biochar [31]. If the O/C ratio was decreased, this suggests that oxygenic functional groups (carbonyl and carboxyl) were reduced, which was in favor of improving biochar stability [33]. As shown in Table S2, the O/C ratio of SDMBC600 was reduced to 0.85 from 0.91 of SDBC600, while the (N+O)/C ratio of SDMBC600 was reduced to 0.97 from 1.04 of SDBC600. The results showed that SDMBC600 was more stable with higher aromaticity and lower polarity than SDBC600.

FTIR had been applied for determining the functional groups of SDBC600 and SDMBC600 (see Figure 1e). The results indicated that the main functional groups of SDBC600 were −OH, O=C−O, C−O−C, C−H, and Si−O−Si. The peaks of Fe−O (588 cm^−1^) and Zn−O (618 cm^−1^) of SDMBC600 appeared after the modification with FeCl_3_/ZnCl_2_. The O=C−O antisymmetric stretching vibration (1462 cm^−1^) and C−H (900 cm^−1^) disappeared, while the intensity of C−O−C (1082 cm^−1^) decreased significantly. These results indicated that Fe-Zn oxides loading on biochar resulted in the change of its functional groups. After the second calcination, the functional group C=C (1662 cm^−1^) of SDMBC600 was enhanced, suggesting that the stability of modified biochar (SDMBC600) was significantly improved.

In the XRD analyses of SDBC600, the diffraction peak at 26.60° belonged to the typical graphite carbon structure (see Figure 1f). The peaks at 26.64° and 36.55° were matched to the characteristic peaks of the (011) and (110) planes of quartz (SiO_2_, PDF 99–0088). Peaks at 23.06°, 29.40°, 35.97°, 39.41°, 43.162°, 47.50°, and 48.51° were matched to the characteristic peaks of the (012), (104), (110), (113), (202), (018), and (116) planes of calcite (CaCO_3_, PDF 99–0022). For SDMBC600, the peak at 35.43° matched the characteristic peaks of the (311) plane of Fe_3_O_4_ (PDF 99–0073), and the peaks at 32.56° and 36.25° matched the characteristic peaks of the (104) plane of ZnCO_3_ (PDF 99–0095) and the (101) plane of ZnO (PDF 99–0111), respectively. The above results also indicated that Fe-Zn was chemically loaded onto SDMBC600.

### 2.2. Adsorptive Processes

#### 2.2.1. Adsorption Kinetics

**Figure 2:**
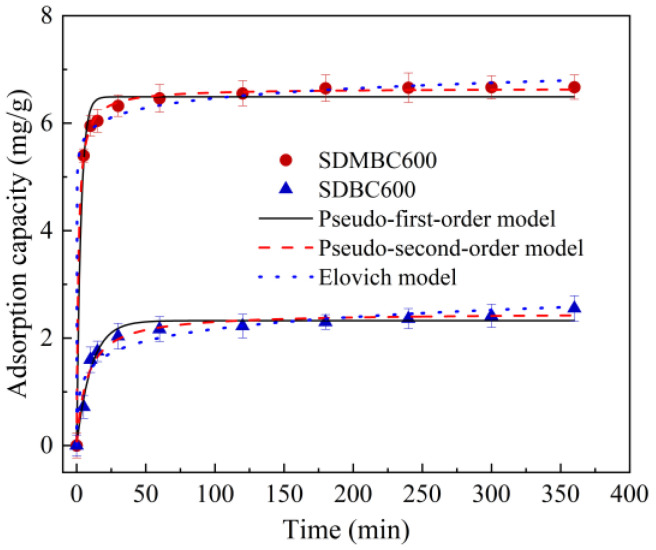
The adsorption kinetics of diuron by SDBC600 and SDMBC600 (5 mg/L diuron, 0.075 g SDMBC600, *t* = 0–360 min).

Figure 2 displays the kinetic adsorption curve of SDBC600 and SDMBC600. The adsorption capacity of diuron by SDBC600 and SDMBC600 achieved an adsorption balance within 6 h, with adsorption capacities of 2.55 and 6.67 mg/g, respectively. The diuron adsorption capacity of SDMBC600 was 2.62 times that of SDBC600, which is likely due to its richer pore structure, greater specific surface area, and more adsorption sites (Table S1). The results showed that SDMBC600 had an enhanced removal efficiency and ability of diuron.

Three adsorption kinetic models have been applied for matching the diuron adsorption by SDBC600 and SDMBC600. In contrast to the other two models (Table 1), the pseudo-second-order kinetic model was best fitted to the adsorption behavior of the biochar, especially for SDMBC600. Most likely, the adsorption of diuron by SDMBC600 was mainly by chemisorption [34]. It was reported that the chemisorption was represented by the Elovich model, which assumed that the surface of the adsorbent was heterogeneous [35]. In the present study, the *R*^2^ > 0.99 in the fitted data parameters of the Elovich model, so supporting the assumption that the rate-controlling process for the adsorption of diuron onto SDMBC600 was chemisorption [36].

#### 2.2.2. Adsorption Isotherms

**Figure 3:**
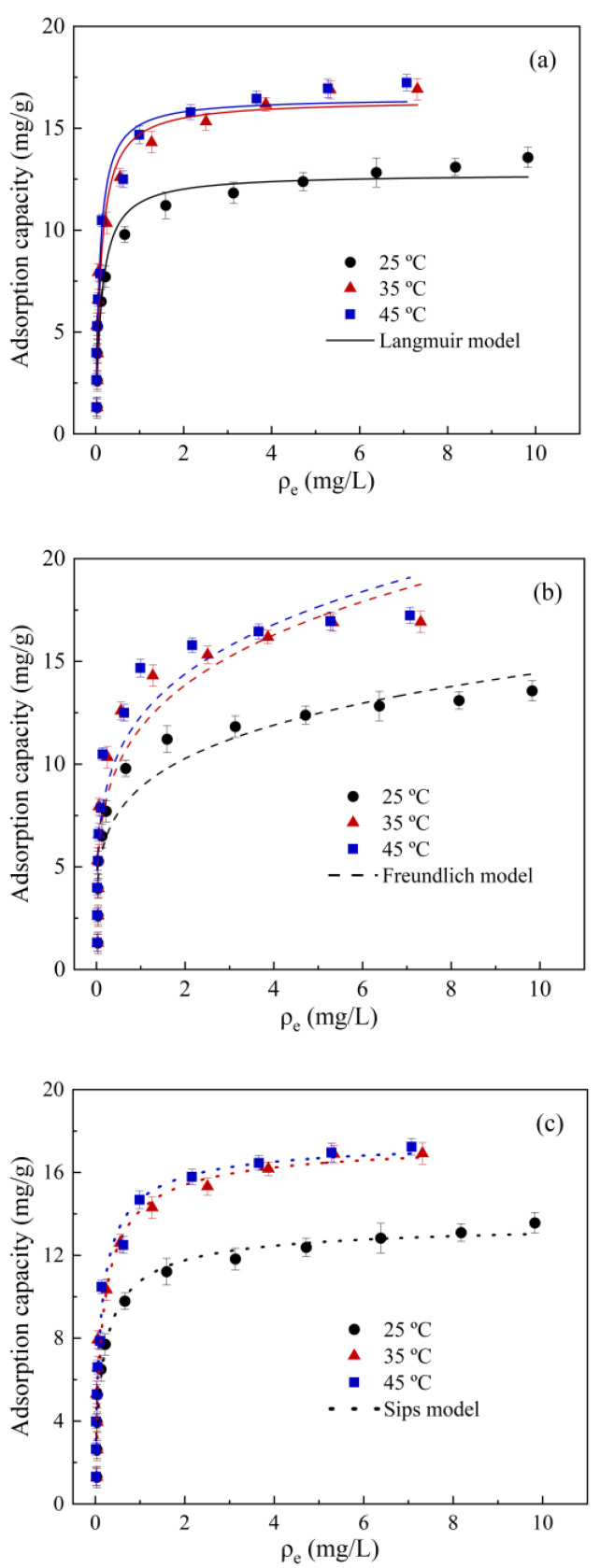
Isotherm model fitting of diuron adsorption by SDMBC600: (**a**) Langmuir model; (**b**) Freundlich model; (**c**) Sips model.

Figure 3 shows the adsorption isotherms of diuron on SDMBC600, and Table 2 shows the parameters obtained by adsorption isotherm fitting. Compared with the Freundlich model, the Langmuir model better expressed the adsorption behavior. The high correlation coefficient (*R*^2^ > 0.93) of the Langmuir model suggests that the adsorption of diuron by SDMBC600 was more consistent with the surface adsorption process [37]. The maximal adsorption capacity of diuron by SDMBC600 could reach 17.7 mg/g by fitting the isotherms model. The SDMBC600 had a better adsorption capacity for diuron than previously reported Sunflower Husks Biochar (0.94 mg/g) and Goethite (0.59 mg/g) [38]. It was also higher than the adsorption capacity of magnetic biochar (11.6 mg/g) prepared by Liu et al. [20] through co-pyrolysis of zero-valent iron nanoparticles and sewage sludge. In addition, based on the fitting data of the Sips model (*R*^2^ > 0.94), the adsorption of diuron by SDMBC involved not only surface adsorption but also a variety of adsorption sites.

### 2.3. Factors Affecting Diuron Adsorption

#### 2.3.1. Effects of Diuron Concentration and Solution Temperature

**Figure 4:**
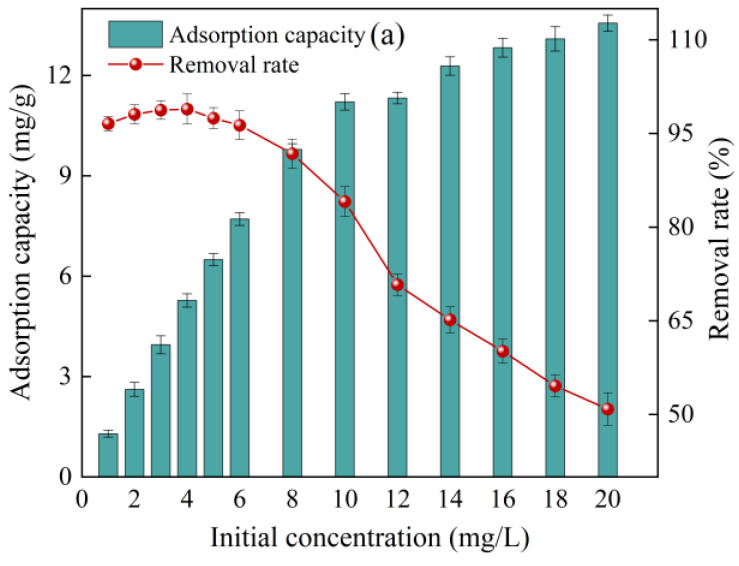

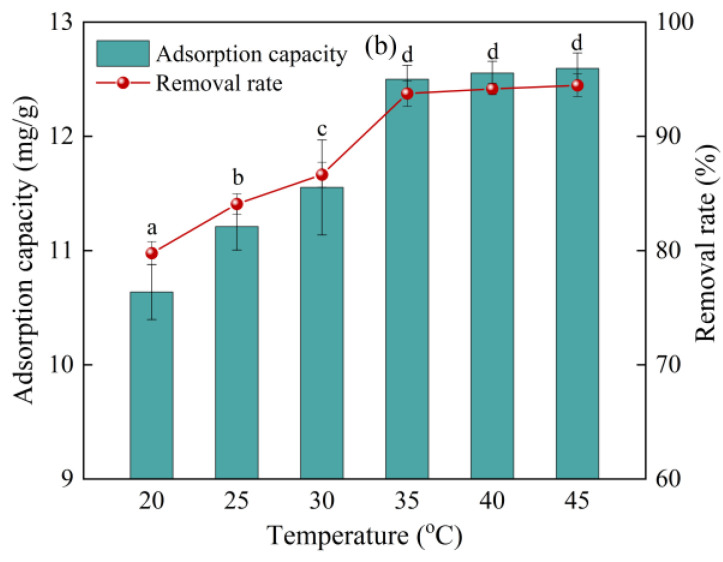
Effects of initial diuron concentration and solution temperature on the adsorption of diuron by SDMBC600: (**a**) initial diuron concentration; (**b**) solution temperature. (0.075 g SDMBC600, *t* = 360 min). Different letters indicate significant differences between different treatments at the 0.05 level, testing by one-way ANOVA.

The adsorption capacity of diuron on the SDMBC600 gradually increased with the increasing initial concentration of diuron (see Figure 4a). This might be attributed to the fact that SDMBC600 has a large pore, a large surface area, and abundant adsorption sites at the initial adsorption stage. Under the action of the concentration difference driving force, diuron molecules continuously migrated and diffused from the aqueous solution to the SDMBC600 surface, and adsorbed on SDMBC600 [24]. However, the adsorption performance showed no obvious change when the initial concentration of diuron was greater than 16 mg/L, most likely because the limited adsorption sites had already been occupied by diuron molecules when the diuron concentration after this concentration. The concentration difference between the remaining diuron molecules in solution and those adsorbed on the SDMBC600 surface continued to decrease, resulting in a slow decrease in the adsorption rate [39]. Yan et al. [24] studied the removal of tetracycline by ZnCl_2_ modified biochar and found that the adsorption capacity of tetracycline was gradually increased as the concentration of tetracycline increased, but no further adsorption could be achieved as the tetracycline concentration was above 80 mg/L.

The adsorption efficiency of diuron by SDMBC600 gradually increased as the solution temperature increased (Figure 4b). This may be explained by the increased diffusion rate of diuron in water at higher solution temperature conditions. This increased the diffusion of diuron molecules to the surface of SDMBC600, thereby promoting the adsorption of diuron on SDMBC600 [40]. In addition, the pore diffusion rate of diuron in SDMBC600 was likely accelerated with higher temperatures, which also promoted the adsorption process [41]. Since SDMBC600 has a good adsorption capacity at 25 °C, this temperature was chosen for the subsequent batch adsorption experiments (such as pH and coexisting substances).

#### 2.3.2. Effects of Solution pH and Coexisting Substances

**Figure 5:**
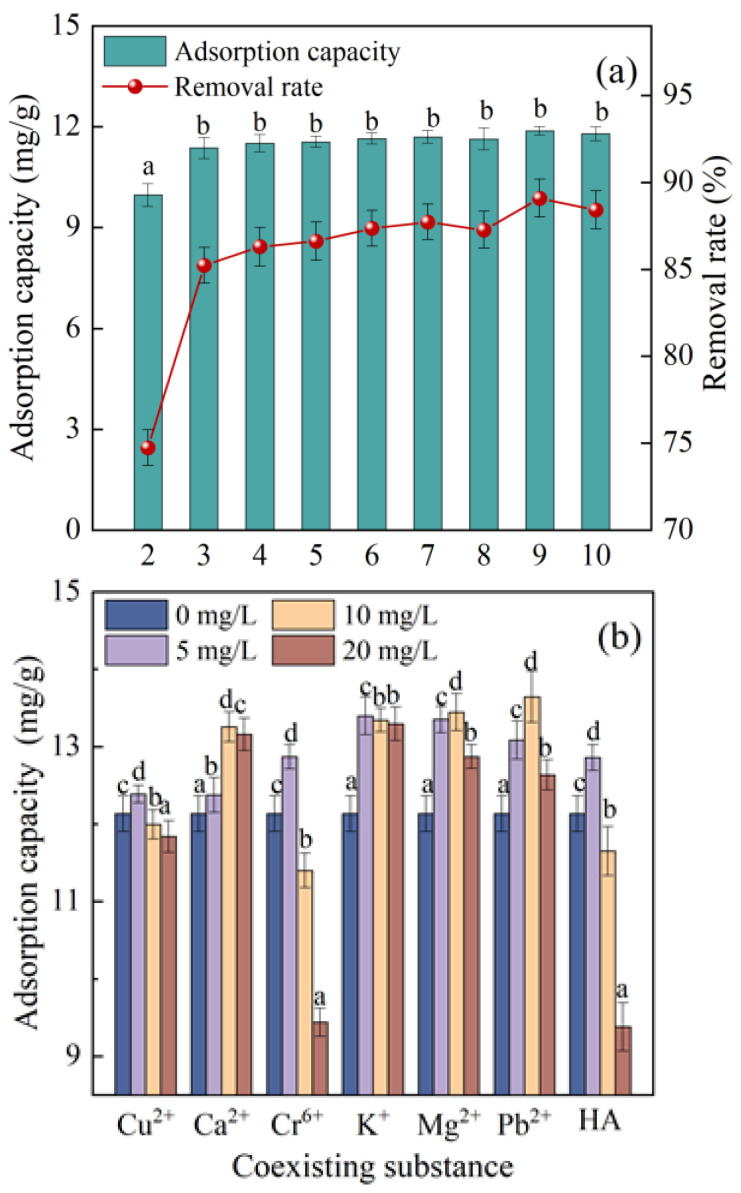
Effects of initial solution pH and coexisting substances on the adsorption of diuron by SDMBC600: (**a**) initial solution pH; (**b**) coexisting substances. (10 mg/L diuron, 0.075 g SDMBC600, *t* = 360 min). Different letters indicate significant differences between different treatments at the 0.05 level, testing by one-way ANOVA.

At a pH of 2, the adsorption capacity of SDMBC600 for diuron was low (Figure 5a), which seems to be caused by the disruption of the structure of SDMBC600 under strong acidic solution conditions. The pH values showed an insignificant effect on the adsorption of diuron by SDMBC600 when the pH of the aqueous solution was greater than 3. This indicates that it could be used in a wide pH range. Note that the adsorption property of SDMBC600 was different from previous studies on the effect of pH. For example, Zhuo et al. [42] studied the performance and mechanism of the simultaneous adsorption of phosphate and tetracycline by calcium modified corn straw biochar. The removal efficiency of phosphate and TC was significantly affected by pH value. However, the present study showed that the initial pH of the solution had no significant effect on the adsorption of diuron by SDMBC600. This indicates that SDMBC600 has an adsorption capacity applied in a wide range of pH. This is likely because SDMBC600 has a strong pH buffering capacity. It was found that, regardless the initial solution was acidic or alkaline, the pH of the solution after adsorption treatment could remain to be neutral when SDMBC600 was used as an adsorbent. In addition, due to the low pK_a_ (−1 to −2) of diuron, the electrostatic action had little influence on the adsorption of diuron. Therefore, SDMBC600 as an adsorbent used for water treatment has good adaptability to solution pH.

Metal ions and natural organic matter such as humic acids (HA) are often present in real water samples, which may interfere with the adsorption of diuron. Considering the typical coexisting substances in natural water bodies and referring to the previous literature [30,43], six metal ions (Cu^2+^, Ca^2+^, Cr^6+^, K^+^, Mg^2+^, Pb^2+^) and HA were selected as coexisting substances in this study to investigate the adsorption and removal of diuron (initial concentration of 10 mg/L) by SDMBC600 (see Figure 5b). It was found that the adsorption of diuron by SDMBC600 was inhibited only in the presence of high concentrations of Cu^2+^, Cr^6+^, and Pb^2+^ (>5 mg/L). This may be due to the competitive adsorption between metal ions and diuron [44]. Another reason may be that ions enter the diffusion bilayer on the surface of SDMBC600, which reduces the repulsive force between biochars. This result is in the formation of a denser aggregate structure for the biochar, which is not favorable for diuron adsorption [45]. Interestingly, in the presence of Ca^2+^, K^+^, Mg^2+^, and low concentrations of Cu^2+^, Cr^6+^, and Pb^2+^ (<5 mg/L), the adsorption of SDMBC600 to diuron was promoted. This may be due to the salting-out effect, which reduces the solubility of the diuron and promotes the diffusion of the diuron on the surface of SDMBC600, thus increasing the adsorption capacity of the diuron on SDMBC600 [46]. In the study, the inhibition rates of metal ions on diuron adsorption ranged from 2.53% to 23.16%. Compared with the previous studies by Nguyen et al. [33] (50%) and Cao et al. [44] (>80%), SDMBC600 had better environmental adaptability and ionic interference resistance. This result indicated that SDMBC600 could adapt certain interferences from metal ions during the adsorption of diuron.

The adsorption capacity of diuron by SDMBC600 was 11.6, 12.4, 11.2, and 8.88 mg/g when the concentration of HA in aqueous solution was 0, 5, 10, and 20 mg/L, respectively (Figure 5b). The adsorption capacity of diuron on SDMBC600 increased (6.24%) when 5 mg/L of HA was added into diuron solution. This is likely because HA itself also has certain adsorption effects [47]. Hence, diuron may be attached with HA and jointly adsorbs on SDMBC600. However, the adsorption capacity of SDMBC600 for diuron decreased at the HA concentration above 5 mg/L. This occurred most likely because HA competed with diuron for adsorption sites at the high concentration on SDMBC600 [48].

### 2.4. Adsorption Mechanism

The adsorption mechanisms of biochar could be explored through its physical and chemical properties [49]. Firstly, compared with SDBC600, SDMBC600 has a greater specific surface area and a higher number of mesopores. The experimental results suggest that the co-modification of Fe-Zn could significantly enhance the pore expansion and specific surface area, thereby enhancing the adsorption of diuron. Secondly, with the decrease of (N+O)/C and H/C in SDMBC600, the aromatic compounds in SDMBC600 were increased, which enhanced the π–π binding between SDMBC600 and diuron [30].

The surface morphology and surface chemical composition of SDMBC600 after adsorption were evaluated by SEM-EDS (see Figure S2). The pores on the surface of SDMBC600 were reduced in size and the surface was loaded with fine particles. This implies that diuron molecules may be adsorbed on the surface of SDMBC600 through pore-filling. The EDS results of SDMBC600 showed an increase in the C and N contents, which further confirmed the attachment of diuron molecules onto the surface of SDMBC600.

Except for pore-filling, the functional groups on SDMBC600 might contribute to the adsorption of diuron. Comparing the FTIR spectra of SDMBC600 before and after the adsorption of diuron, the interaction between biochar and diuron could be inferred. The FTIR spectrum of SDMBC600 after adsorption can be seen in Figure S2. The peak of C=C in the FTIR spectra of SDMBC600 shifted from 1662 to 1673 cm^−1^ after the adsorption of diuron, which indicated that π–π binding was involved in diuron adsorption [50]. The peak of –OH shifted from 3502 to 3522 cm^−1^ in SDMBC600 and its intensity increased evidently, indicating that the hydrogen bond was involved in the adsorption of diuron [51]. Moreover, the Fe–O and Zn–O peaks in the FTIR spectra of SDMBC600 moved and their intensity decreased after the adsorption of diuron, providing evidence for surface complexation [31].

**Figure 6:**
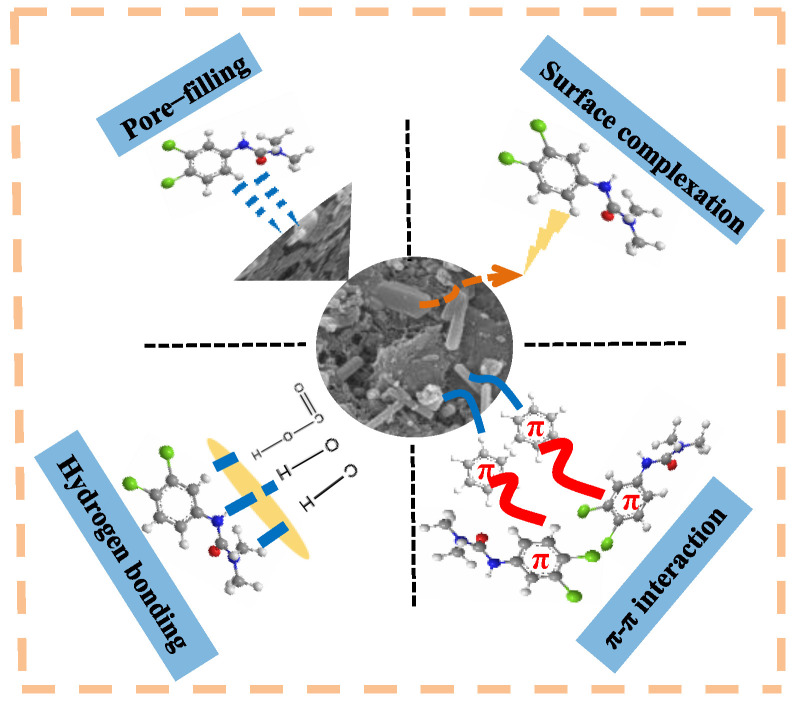
Schematic diagram of the adsorption mechanism of biochar to diuron.

Overall, the adsorption mechanism of diuron by SDMBC600 included surface complexation, π–π binding, hydrogen bonding, and pore filling (Figure 6).

### 2.5. Practical Research

#### 2.5.1. Adsorption of Pesticide Mixture in Solution

**Figure 7:**
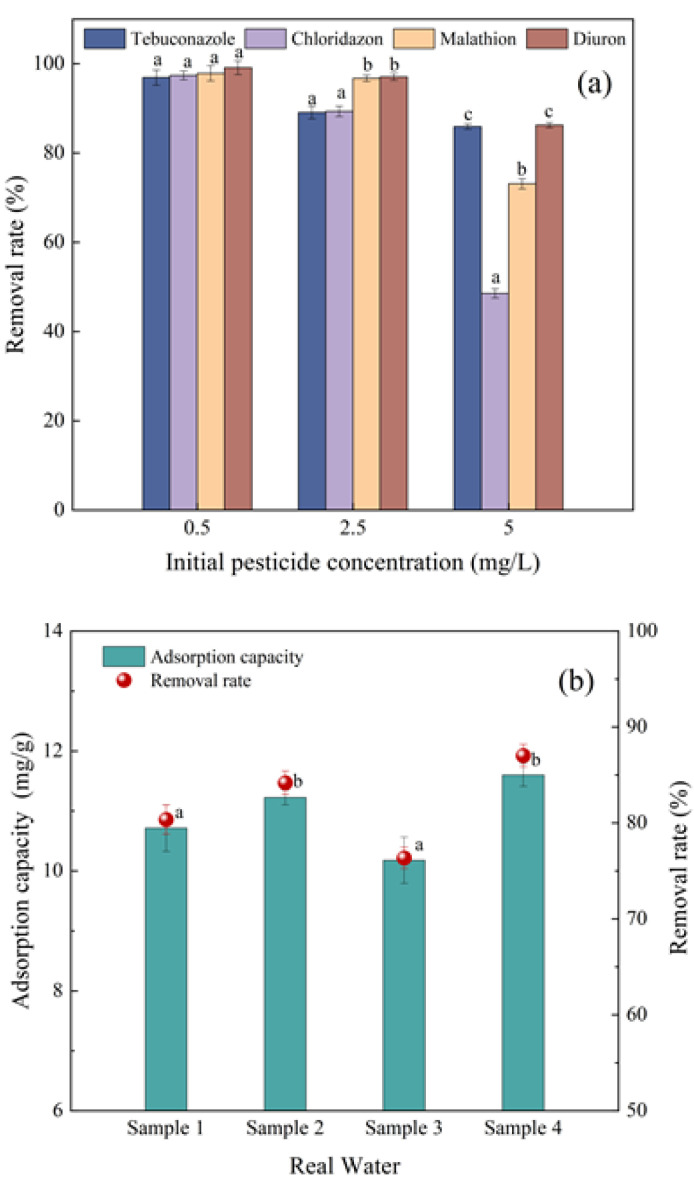
Adsorption capacity of SDMBC600 in mixed pesticide solution and real water sample: (**a**) mixed pesticides solution (tebuconazole, chloridazon, malathion, and diuron); (**b**) real water sample. (10 mg/L diuron, 0.075 g SDMBC600, *t* = 360 min). Different letters indicate significant differences between different treatments at the 0.05 level, testing by one-way ANOVA.

In order to explore the adsorption efficiency of biochar for a variety of target pollutants, the adsorption effect of SDMBC600 on four mixed pesticides was studied (Figure 7a). The removal rates of 0.5 mg/L mixed pesticides solution (tebuconazole, chloridazon, malathion, and diuron) by SDMBC600 were fairly high, and the removal rates of the four pesticides were 96.92%, 97.38%, 97.87%, and 99.11%, respectively. The removal rates for the four pesticides by SDMBC600 decreased while the concentration of pesticides increased (0.5–5 mg/L), which may be caused by competitive adsorption among the four pesticides. It is worth noting that SDMBC600 showed a higher removal rate for diuron than the other pesticides. This was because diuron was a large molecule with multiple non-polar functional groups and an aromatic ring structure, and the high electron density of the benzene ring in diuron enhanced its dispersion force. The stronger the effect of the molecular dispersion force of pesticide, the easier it can absorb on adsorbent [52]. In conclusion, SDMBC600 had a better adsorption performance for the different organic pollutants.

#### 2.5.2. Adsorption in Real Water

The adsorption efficiency for diuron in real water solutions by SDMBC600 was also studied (Figure 7b, physicochemical properties of the real water samples are shown in Table S3). In ultrapure water, the adsorption capacity of SDMBC600 for diuron was 11.1 mg/g, whereas, in the real water samples of 1 to 4, the adsorption capacities of diuron were 10.7, 11.2, 10.2, and 11.6 mg/g, respectively. The results showed that water samples 2 and 4 promoted the adsorption of diuron by SDMBC600, while water samples 1 and 3 inhibited the adsorption of diuron by SDMBC600. This was likely due to the higher total organic carbon (TOC) in the real water samples 1 and 3, with the presence of more organic aromatic compounds in the two water samples, they would compete with diuron in solution to reduce the adsorption capacity of diuron on SDMBC600 [44]. Secondly, according to the results of the coexistence ion experiment above, the presence of calcium ions in solution could promote the adsorption of diuron by SDMBC600. This indicates that the real water samples matrix had some insignificant influence on the adsorption of diuron by SDMBC600. In general, SDMBC600 can still achieve good adsorption efficiency for diuron under real water quality conditions. According to the above research, SDMBC600 also has certain adsorption capacity in the actual wastewater.

#### 2.5.3. Stability and Regeneration

In order to explore the safety of SDMBC600 in practical applications, the leaching concentrations of iron and zinc were measured at pH 3, 5, 7, and 10 (see Figure S3). The results showed that the leaching concentrations of iron and zinc decreased with the increase of solution pH value. When the pH value among 5 to 10, the leaching concentration of Fe (0.0525–0.203 mg/L) and Zn (0.00975–0.0310 mg/L) was lower than the limits for the concentration of 0.3 mg/L and 1.0 mg/L in the Standards for Drinking Water Quality in China (GB5749-2022), respectively. Only when the pH value was 3, was the leaching concentration of Fe greater than 0.3 mg/L. It showed that SDMBC600 was chemically stable and environmentally safe in a wide pH range.

Sorbent regeneration could give biochar a low-cost advantage. The regeneration combination of ultrasonic and organic solvents (methanol, ethanol, acetone, etc.) is an effective adsorbent regeneration method. In this research, SDMBC600 was regenerated by ultrasonic and ethanol co-treatment. SDMBC600 could be effectively regenerated by ultrasonic-ethanol co-treatment and maintain good regeneration adsorption performance during the repeated adsorption cycle. The adsorption capacity at four adsorption–regeneration cycles is still 99.6% of the first adsorption capacity (Figure S4). The regeneration effect of SDMBC600 is better than that of previous studies (44.5% and 76.25%) [30,44]. It can be seen that the adsorption capacity of the first three regeneration cycles of SDMBC600 was better than that of the new preparation SDMBC600. This might be caused by the fact that ultrasound could open the pore size and ethanol could change the surface functional groups of SDMBC600. Similar results had been obtained in relevant studies [53]. The results showed that SDMBC600 could still achieve an ideal regeneration property in multiple regeneration cycles through ultrasonic and ethanol co-treatment.

## 3. Materials and Methods

### 3.1. Reagents and Materials

Municipal excess-activated sewage sludge was obtained from a sewage treatment plant in Yantai City (Shandong Province, China). Diuron (>99.6%) and Chloridazon (>97%) were supplied by J&K Scientific Ltd. (St. Louis, MO, USA). Tebuconazole (>95%) and Malathion (>99.7%) were purchased from Sigma-Aldrich Corporation (Bellefonte, PA, USA). Methanol (HPLC grade) and acetonitrile (HPLC grade) were purchased from Sigma-Aldrich Corporation (Bellefonte, PA, USA). The other chemical reagents (analytical grade) were purchased from Sinopharm Chemical Reagent Co., Ltd. (Shanghai, China) and used without further purification. Considering the diversity and representativeness of the real water quality, water samples 1 to 4 were taken from Yantai City (Shandong Province) and were samples of the Guangdang River, the Fenghuangshan Reservoir, the Miaohou Reservoir, and tap water, respectively (water quality indexes of the four samples are shown in Table S3).

### 3.2. Preparation of Adsorbent

The municipal excess-activated sewage sludge was dried for 6 h in an electrically heated thermostatic drying oven (101-1BS) at 80 °C to remove moisture. The dried sludge was placed in a vacuum pipe furnace (SK-B05123K-200) filled with N_2_ and raised to 600 °C at a rate of 10 °C/min. The biochar (named after SDBC600) was obtained after two hours of pyrolysis treatment under nitrogen protection.

Modified biochar was prepared by two–step pyrolysis. The FeC1_3_, ZnC1_2_, and SDBC600 were added to 500 mL of ultrapure water in a mass ratio of 0.45:0.25:1 (2.25 g:1.25 g:5 g) (see Figure S5 for proportion selection). The mixed solution was treated with ultrasound for 30 min and then stirred at 60 °C for 12 h. The solid–liquid phase in the mixed solution was separated and dried (80 °C, 3h). The sample obtained in the previous step was calcination in a vacuum pipe furnace at 600 °C for 40 min to obtain the modified biochar (named SDMBC600), which was washed with hydrochloric acid to remove the ash in SDMBC600 (the optimum pyrolysis temperature of the second step was obtained by pre-experiment. see Figure S6). Then, ultrapure water was used to wash the SDMBC600 until the washed water was neutral.

### 3.3. Characterization of the Adsorbent

The surface morphology of biochar was analyzed with a Scanning Electron Microscope-Energy Dispersive Spectrometer (SEM-EDS, Carl Zeiss AG, Sigma 500, Darmstadt, Germany). The levels of H, C, N, O, and S were evaluated through an Elemental Analyzer (EA, Elementar, Vario EL Cube, Langenselbold, Germany). Fourier Transform Infrared Spectroscopy (FTIR, Thermo fisher, Nicolet iS5, Waltham, MA, USA) was used to determine the functional groups of biochar. Brunauer-Emmett-Teller (BET, Micromeritics, ASAP2460, Norcross, GA, USA) was employed to examine the N_2_ adsorption at 77 K, using a fully automatic fast specific surface and porosity analyzer. The X-ray diffraction (XRD, Rigaku, Smart Lab 9, Tokyo, Japan) of biochar was collected over a range of 10–90°.

### 3.4. Adsorption Experiments

The effects of the initial concentration of diuron, solution pH, solution temperature, water quality composition (coexisting ions and organic matter), as we as real water samples on the adsorption capacity of modified biochar (SDMBC600) were analyzed by batch experiments. The safety and reusability of modified biochar (SDMBC600) were also investigated. The pH of the diuron solution was adjusted with 0.1 M HCl and NaOH in batch adsorption experiments. The samples were collected at defined intervals and filtered through a 0.22 μm polyether sulfone membrane before quantitative analysis of diuron in aqueous solution by ultra-high performance liquid chromatography-electrospray ionization-triple quadrupole mass spectrometer (UPLC-ESI-MS/MS, ACQUITY UPLC/TQD, Waters, USA, see Text S1 for details of operation parameter). All the experiments were conducted in triplicate and average values and standard deviations were reported. In this study, the experimental parameters and the model equation are summarized in Table 3 and Table S4, respectively. Statistical analyses were performed using the SPSS software (version 20.0).

## 4. Conclusions

In this study, the SDBC600 modified by Fe-Zn showed good adsorption capacity. The adsorption of diuron from water by SDMBC600 conformed to the pseudo-second-order kinetic and the Langmuir isotherm model. SDMBC600 could achieve better adsorption capacity for diuron in a wide pH range. The coexisting metal ions and HA in aqueous solution had only negligible effects on the adsorption of diuron by SDMBC600 (76.3–111%). The main mechanisms of diuron adsorption by SDMBC600 were surface complexation, π–π binding, hydrogen bonding, and pore-filling. In addition, SDMBC600 had also good adsorption capacity in real water conditions and it is structurally stable under water solution conditions. It could be regenerated up to four times, and still maintained 99.6% of the initial adsorption capacity. The excellent adsorption capacity, environmental adaptability, safety, and regeneration properties of SDMBC600 offered the possibility of excess activated sludge resource utilization.

## Figures and Tables

**Table 1:** Adsorption kinetic parameters for diuron by SDBC600 and SDMBC600.

Fitting Model	Parameter	SDBC600	SDMBC600
Experimental adsorption capacity	*q*_m_ (mg/g) ^a^	2.55	6.67
Pseudo-first-order	*q*_m_ (mg/g) ^b^	2.32	6.49
	*k*_1_ (min^−1^)	0.093	0.33
	*R* ^2^	0.973	0.989
Pseudo-second-order	*q*_m_ (mg/g) ^b^	2.4721	6.65
	*k*_2_ (g/(mg·min))	0.0542	0.12
	*R* ^2^	0.975	0.999
Elovich	α (g/(mg·min))	2.67	1.61 × 108
	*β* (g/mg)	3.10	3.84
	*R* ^2^	0.934	0.996

^a^ Observed value; ^b^ Calculated value.

**Table 2:** Fitting parameters of adsorption isotherm for experimental data.

Fitting Model	Parameter	Temperature (°C)		
		25	35	45
Langmuir	*Q*_m_ (mg/g)	12.8	16.4	16.5
	*K*_L_ (L/mg)	7.96	8.68	11.3
	*R* ^2^	0.955	0.935	0.971
Freundlich	*K*_F_ (mg/g(L/mg)^1/n^)	8.87	11.8	12.3
	*n*	4.73	4.27	4.47
	*R* ^2^	0.901	0.878	0.904
Sips	*q*_m_ (mg/g)	13.7	17.6	17.7
	*K*_s_ (L/mg)	5.81	6.26	8.20
	*m*	0.74	0.77	0.75
	*R* ^2^	0.964	0.942	0.979

**Table 3:** Specific experimental parameters of SDMBC600 adsorption of diuron.

Experiment	Parameter
Kinetics	*C*_0_ = 5 mg/L, *V* = 0.1 L, *m* = 0.075 g, *t* = 0–360 min, and *T* = 25 °C
Isotherm	*C*_0_ = 1–20 mg/L, *V* = 0.1 L, *m* = 0.075 g, *t* = 360 min, and *T* = 25/35/45 °C
Diuron initial concentration	*C*_0_ = 1–20 mg/L, *V* = 0.1 L, *m* = 0.075 g, *t* = 360 min, and *T* = 25 °C
Temperature	*C*_0_ = 10 mg/L, *V* = 0.1 L, *m* = 0.075 g, *t* = 360 min, and *T* = 20–45 °C
Solution pH	*C*_0_ = 10 mg/L, *V* = 0.1 L, *m* = 0.075 g, *t* = 360 min, and pH = 2–10
Coexisting ions and HA	*C*_0_ = 10 mg/L, *V* = 0.1 L, *m* = 0.075 g, *t* = 360 min, and the concentration of coexisting ions (Cu^2+^, Ca^2+^, Cr^6+^, K^+^, Mg^2+^, Pb^2+^) and HA were 0–20 mg/L.
Pesticide mixture	*C*_0_ = 0.25/2.5/5 mg/L, *V* = 0.1 L, *m* = 0.075 g, *t* = 360 min, and *T* = 25 °C
Real water	*C*_0_ = 10 mg/L, *V* = 0.1 L, *m* = 0.075 g, *t* = 360 min, and *T* = 25 °C

## Data Availability

Data is contained within the article or the supplementary data file.

## Supplementary Materials

The following supporting information can be downloaded at: https://www.mdpi.com/article/10.3390/molecules28062868/s1, Text S1: Method for the detection of diuron; Figure S1: The N_2_ adsorption–desorption curves of SDBC600 and SDMBC600; Figure S2: Characterization of SDMBC600 after adsorption of diuron from water: (a) SEM image; (b) EDS; (c) FTIR spectrum; Figure S3: The leaching concentrations of Fe and Zn of SDMBC600 at different solution pH conditions (t = 360 min); Figure S4: The regeneration efficiency of SDMBC600 by ultrasonic–ethanol co-treatment (10 mg/L diuron, 0.075 g SDMBC600, *t* = 360 min); Figure S5: The ratio of FeCl_3_, ZnCl_2_, and SDBC600 on the adsorption of diuron by modified biochar; Figure S6: Effect of the second step pyrolysis temperature on the adsorption of diuron by modified biochar; Table S1: Surface area and pore parameters of SDBC600 and SDMBC600; Table S2: Elemental composition and atomic number ratio of the biochars; Table S3: Water quality index of the real water samples; Table S4: Details of the equations, kinetic models, isotherm models, and thermodynamics model used in this study.
