# Supplementary material for: A Stable Fe-Zn Modified Sludge-Derived Biochar for Diuron Removal: Kinetics, Isotherms, Mechanism, and Practical Research
Source: Molecules. 2023 Mar 22;28(6):2868. doi: 10.3390/molecules28062868 (PMC10058066; doi:10.3390/molecules28062868)
Supplement: Supplementary file 1 [file molecules-28-02868-s001.zip › molecules-2281020-supplementary.pdf]

A stable Fe-Zn Modified Sludge-Derived Biochar for Diuron Removal: Kinetics, Isotherms, Mechanism, and Practical Research

Yucan Liu[1], Xianguo Ji[1], Ying Wang[1], Yan Zhang[1], Yanxiang Zhang[2*], Wei Li[3], Jiang Yuan[4], Dong Ma[5], Hongwei Sun[2], Jinming Duan[6]

[1] *School of Civil Engineering, Yantai University, Yantai 264005, China*

[2] *School of Environmental and Materials Engineering, Yantai University, Yantai 264005, China*

[3] *Key Laboratory of Northwest Water Resources, Environment and Ecology, MOE, Xi'an University of Architecture and Technology, Xi'an 710055 China*

[4] *College of Environmental Science and Engineering, Tongji University, Shanghai 200092, China*

[5] *Rural Environmental Engineering Center of Qingdao, College of Resource and Environment, Qingdao Agricultural University, Qingdao 266109, China*

[6] *Centre for Water Management and Reuse, University of South Australia, Mawson Lakes Campus, SA 5095, Australia*

Corresponding author:

E-mail: yanxiangzhang@ytu.edu.cn (Yanxiang Zhang); phone: 86-15699613180

Table of Contents

Text S1 Method for the detection of diuron. 3

Figure S1 The N_2 adsorption–desorption curves of SDBC600 and SDMBC600. 4

Figure S2 Characterization of SDMBC600 after adsorption of diuron from water: (a) SEM image; (b) EDS; (c) FTIR spectrum. 5

Figure S3 The leaching concentrations of Fe and Zn of SDMBC600 at different solution pH conditions (t = 360 min). 6

Figure S4 The regeneration efficiency of SDMBC600 by ultrasonic–ethanol co–treatment (10 mg/L diuron, 0.075 g SDMBC600, t = 360 min). 7

Figure S5. The ratio of $FeCl_3$, $ZnCl_2$, and SDBC600 on the adsorption of diuron by modified biochar. 8

Figure S6 Effect of the second step pyrolysis temperature on the adsorption of diuron by modified biochar. 9

Table S1 Surface area and pore parameters of SDBC600 and SDMBC600. 10

Table S2 Elemental composition and atomic number ratio of the biochars. 11

Table S3 Water quality index of the real water samples. 12

Table S4 Details of the equations, kinetic models, isotherm models, and thermodynamics model used in this study. 13

Text S1 Method for the detection of diuron.

UPLC–ESI–MS/MS was used as a determination and analysis instrument, and Masslynx 4.1 was used for real–time control, data acquisition, and processing. ACQUITYTM UPLC BEH C18 column (2.1 mm × 50 mm × 1.7 μm) was used for chromatographic separation. The mobile phase consisted of MeOH (A) and UPW (B) at a flow rate of 0.2 mL/min and an injection volume of 10 μL. The column temperature was maintained at 35 ºC. The injection syringe was rinsed automatically after each injection to eliminate interference between injections. Each rinse cycle used 800 μL of weak wash solvent (UPW/MeOH, 90:10, v:v) and 400 μL of strong wash solvent (UPW/MeOH, 10:90, v:v). The elution gradient of diuron detection were as follows: 0–1 min, 10% A; 1–5.5 min, 10%–70% A; 5.5–6.5 min, 70%–100% A; 6.5–7 min, 100% A; 7.0–7.1 min, 100%–10% A; 7.1–10 min, 10% A. A total acquisition time of one run analysis was 10 min.

Full scan data were acquired from m/z 50 to 400 at an acquisition rate of 100 ms/scan in both positive electrospray ionization (ESI+) mode and negative electrospray ionization (ESI–) mode. In order to improve the accuracy of the detection, *m*/*z* 232.8 was used for the quantitative analysis of diuron in the selected ion monitoring (SIM, ESI–). The capillary voltage was 3.0 kV and the cone voltage was 34 V. The desolvation and source temperature were 350 ^{o}C and 110 ^{o}C, respectively. The desolvation gas and cone gas were nitrogen (99.999%) with flow rates of 500 L/h and 50 L/h, respectively..

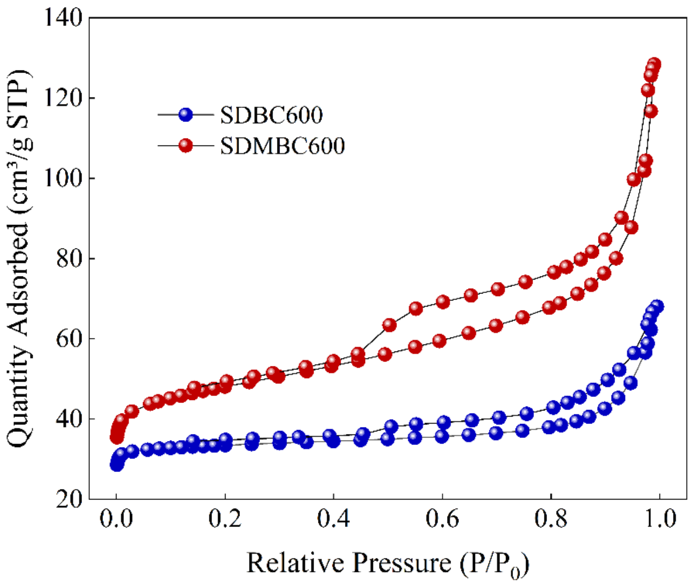

Figure S1 The N_2 adsorption–desorption curves of SDBC600 and SDMBC600.

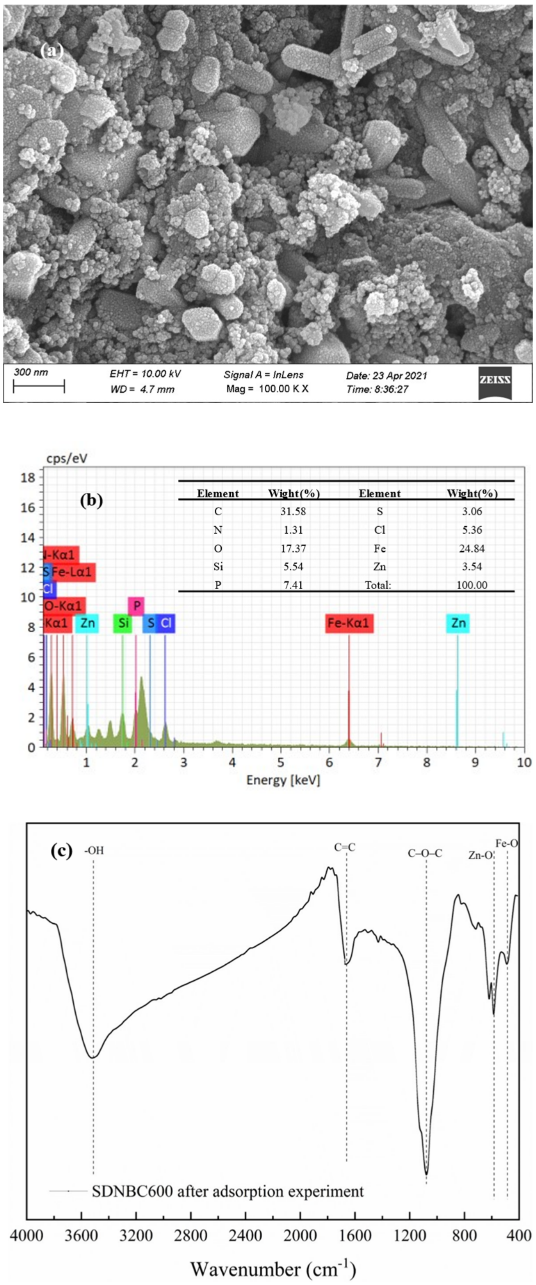

Figure S2 Characterization of SDMBC600 after adsorption of diuron from water: (a) SEM image; (b) EDS; (c) FTIR spectrum.

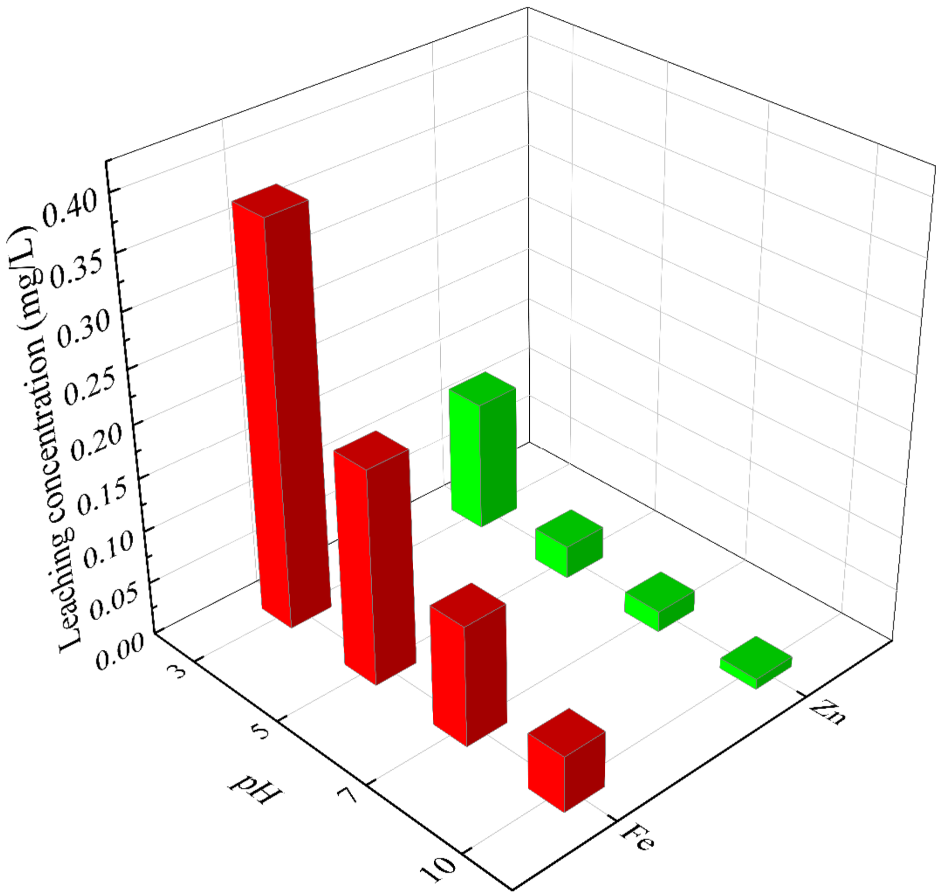

Figure S3 The leaching concentrations of Fe and Zn of SDMBC600 at different solution pH conditions (t = 360 min).

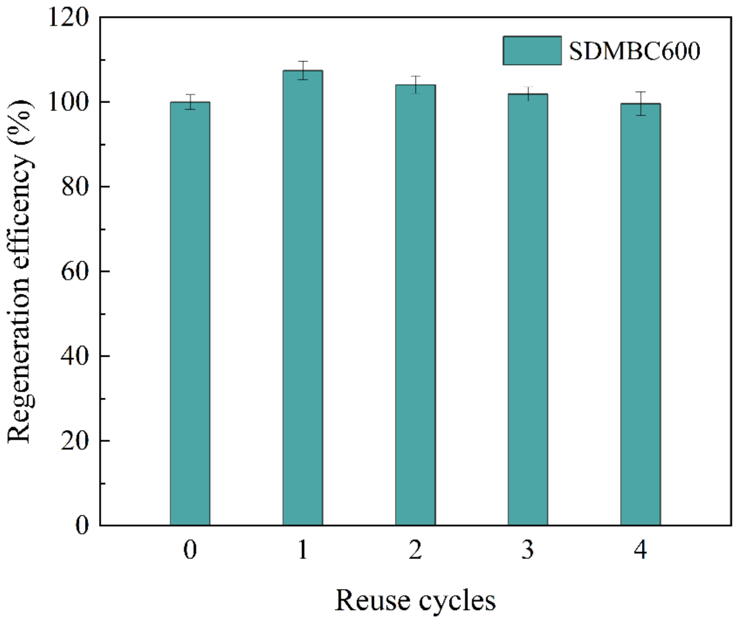

Figure S4 The regeneration efficiency of SDMBC600 by ultrasonic–ethanol co–treatment (10 mg/L diuron, 0.075 g SDMBC600, t = 360 min).

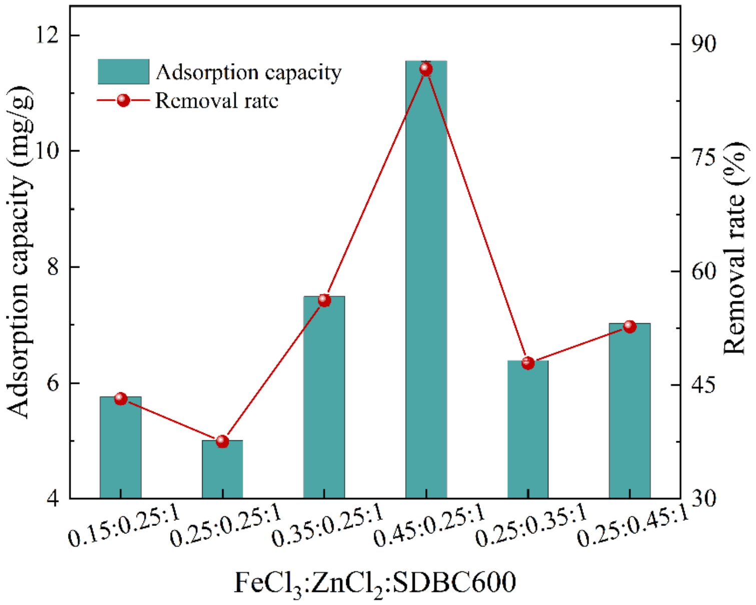

Figure S5. The ratio of $FeCl_3$, $ZnCl_2$, and SDBC600 on the adsorption of diuron by modified biochar.

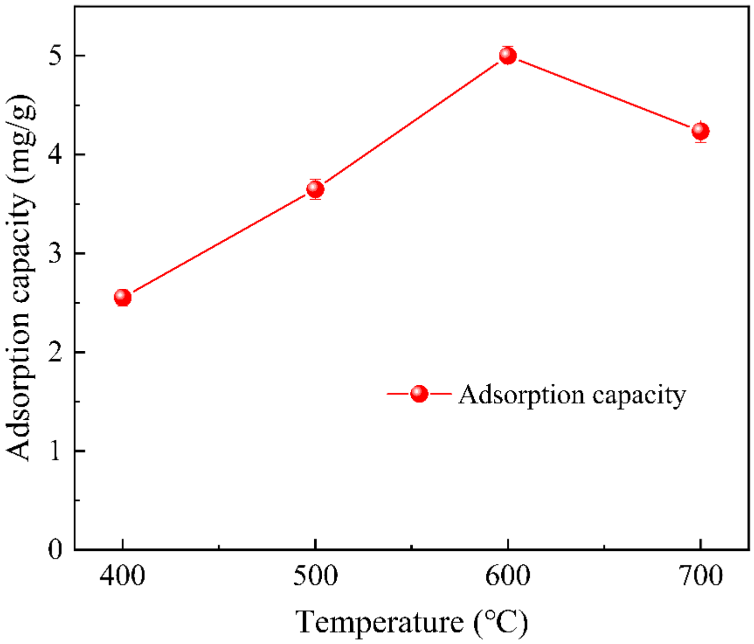

Figure S6 Effect of the second step pyrolysis temperature on the adsorption of diuron by modified biochar.

Table S1 Surface area and pore parameters of SDBC600 and SDMBC600.

Biochar	BET surface area (m^2/g)	Langmuir surface area (m^2/g)	V_{total} (cm^3/g)	V_{micro} (cm^3/g)	Average pore diameter (nm)
SDBC600	28.7	59.8	0.0382	0.00671	5.33
SDMBC600	79.2	204	0.0985	0.00837	4.97

Table S2 Elemental composition and atomic number ratio of the biochars.

Name	Elemental composition (%)					Atomic number ratio (%)		
	H	C	N	S	O	H/C	O/C	(N+O)/C
SDBC600	0.859	22.980	2.880	1.743	20.911	0.116	0.910	1.035
SDMBC600	1.072	20.620	2.460	1.615	17.218	0.166	0.852	0.974

Table S3 Water quality index of the real water samples.

Parameter	Samples 1	Samples 2	Samples 3	Samples 4
Diuron (μg/L)	0.11±0.001	0.008±0.0001	0.22±0.005	ND[a]
Turbidity (NTU)	11.34±0.32	10.22±0.18	5.43±0.11	0.14±0.003
pH	7.35±0.18	8.27±0.22	8.18±0.32	7.94±0.31
TOC (mg/L)	11.14±0.35	4.86±0.13	13.49±0.75	1.24±0.09
TC (mg/L)	33.89±1.23	32.87±1.11	53.71±2.13	35.71±1.52
IC (mg/L)	22.75±1.03	28.01±1.08	40.36±2.31	34.47±1.75
UV_{254} (cm^{-1})	0.18±0.09	0.086±0.001	0.19±0.085	0.02±0.003
NH_3-N (mg/L)	1.835±0.07	0.065±0.001	0.064±0.003	0.003±0.0001
Nitrate Nitrogen (mg/L)	0.38±0.005	2.31±0.12	6.65±0.24	0.98±0.08
Nitrite Nitrogen (mg/L)	0.26±0.002	0.008±0.0001	0.005±0.0001	0.005±0.0003
TN (mg/L)	4.27±0.84	2.95±0.39	7.16±0.99	0.98±0.09
Phosphate (mg/L)	0.160±0.03	0.103±0.02	0.294±0.06	0.014±0.001
TP (mg/L)	0.27±0.08	1.034±0.11	0.34±0.009	0.09±0.001
Chloride (mg/L)	279.20±3.23	155.80±2.51	213.50±3.15	128.30±2.64
Sulfate (mg/L)	263.40±2.64	195.41±1.94	198.82±1.98	113.20±1.67
Total Hardness (Calculated by $CaCO_3$, mg/L)	213.51±3.42	300.57±4.26	232.51±3.21	420.14±4.89

[a] Not detected.

Table S4 Details of the equations, kinetic models, isotherm models, and thermodynamics model used in this study.

Name	Equation	Parameter
Adsorption capacity	$q_e = \frac{C_0 - C_e}{m} V$	q_e was the equilibrium adsorption capacity (mg/g); C_0 was the initial concentration of diuron (mg/L); C_e was the equilibrium residual concentrations of diuron (mg/L); V was the total volume of the solution (L); m was adsorbent mass (g)
Removal rate	$R_e = \frac{C_0 - C_e}{C_0} \times 100\%$	R_e was the removal rate (%); C_0 was the initial concentration of diuron (mg/L); C_e was the equilibrium residual concentrations of diuron (mg/L).
Langmuir	$q_e = \frac{q_m K_L \rho_e}{1 + K_L \rho_e}$	q_e was the equilibrium adsorption capacity (mg/g); ρ_e was equilibrium solution concentration (mg/L); q_m was the maximum adsorption capacity (mg/g); K_L was Langmuir parameter
Freundlich	$q_e = K_f \rho_e^{\frac{1}{n}}$	q_e was the equilibrium adsorption capacity (mg/g); K_f and n were Freundlich parameter; ρ_e was equilibrium solution concentration (mg/L).
Sips	$q_e = \frac{q_m (k_s \rho_e)^m}{1 + (k_s \rho_e)^m}$	q_e was the equilibrium adsorption capacity (mg/g); q_m was the maximum adsorption capacity (mg/g); k_s and ρ_e were Sips model parameter; m was the empirical parameter of Sips.
Pseudo-first-order	$q_t = q_e(1 - e^{-k_1 t})$	q_t was the adsorption capacities (mg/g) at t time (min), q_e was the adsorption capacities (mg/g) at equilibrium time (min); k_1 was the reaction rate constant of the Pseudo-first-order kinetic equation
Pseudo-second-order	$q_t = \frac{{q_e}^2 k_2 t}{1 + q_e k_2 t}$	q_t was the adsorption capacities (mg/g) at t time (min), q_e was the adsorption capacities (mg/g) at equilibrium time (min); k_2 was the reaction rate constant of the Pseudo-second-order kinetic equation
Elovich	$q_t = \frac{ln(1 + \alpha\beta t)}{\beta}$	q_t was the adsorption capacities (mg/g) at t time (min), q_e was the adsorption capacities (mg/g) at equilibrium time (min); α and β were Elovich parameter
